# Postoperative pain outcomes following minor oral surgery in opioid tolerant patients: a retrospective cohort study

**DOI:** 10.1007/s00784-026-07229-4

**Published:** 2026-10-21

**Authors:** Adarsh Gopinath, Femi E. Ayeni, Stephen Cox

**Affiliations:** 1 Oral Surgery Department, Oral Health Service, Nepean Hospital, Penrith, NSW Australia; 2 Nepean Institute of Academic Surgery, Nepean Clinical School, The University of Sydney, 62 Derby St., Sydney, Kingwood, NSW 2747 Australia; 3 Faculty of Medicine and Health, Sydney Dental School, The University of Sydney, Sydney, NSW Australia; 4 Western NSW Oral Health Service, Dubbo, NSW Australia

**Keywords:** Opioid-Related Disorders, Opioid tolerance, Analgesics, Opioid, Postoperative Pain, Oral Surgery, Analgesia

## Abstract

**Background:**

Evidence guiding postoperative pain management in patients taking prescription or illicit opioids undergoing minor oral surgery is limited. It remains unclear whether standard analgesic protocols for opioid naïve patients provide adequate pain relief in opioid tolerant individuals. This study evaluated whether standard postoperative analgesia achieves adequate pain control in opioid tolerant patients undergoing minor oral surgery with expected mild to moderate pain.

**Methods:**

A single centre retrospective study was conducted of patients undergoing minor oral surgical procedures at a public oral surgery service between May 2020 and May 2024. Patients were classified as opioid tolerant if prescribed opioids for longer than one month, receiving methadone therapy, reporting current illicit opioid use, or having a documented history of opioid use disorder. Opioid naïve patients served as controls. Standard postoperative analgesia consisted of paracetamol and non-steroidal anti-inflammatory drugs unless contraindicated. The primary outcome was return with postoperative pain requiring additional opioid analgesia.

**Results:**

A total of 1,263 patient records met inclusion criteria, including 184 opioid tolerant and 1,079 opioid naïve patients. Opioid tolerant patients were significantly more likely to return with postoperative pain requiring additional opioid analgesia compared with opioid naïve patients (28.8% vs. 2.2%; adjusted OR 24.59, 95% CI: 13.26–45.62; *p* < 0.001). This association remained consistent across demographic subgroups and extraction types.

**Conclusions:**

Current post operative analgesic pathways may not fully address the analgesic requirements of some opioid exposed patients. This study demonstrates that opioid tolerance is associated with an increased risk of return presentation requiring additional opioid analgesia.

**Clinical relevance:**

Opioid tolerance is associated with an increased risk of inadequate postoperative pain control, highlighting the need for tailored analgesia beyond standard regimens.

## Introduction

Effective postoperative pain management is central to safe and satisfactory oral surgical care. For most patients undergoing minor oral surgical procedures, postoperative pain can be adequately managed using non-opioid analgesics such as paracetamol and non-steroidal anti-inflammatory drugs (NSAIDs) [1–3]. However, these recommendations are derived almost exclusively from opioid naïve populations. An increasing number of patients presenting for oral surgery are opioid tolerant due to chronic opioid exposure, opioid use disorder, or opioid substitution therapy [4–7]. Opioid tolerance alters pain perception and analgesic responsiveness through receptor downregulation and neuroplastic changes and may be compounded by opioid-induced hyperalgesia [8–10].

In non-dental surgical disciplines, opioid tolerance has been associated with poorer postoperative pain outcomes and increased analgesic requirements [4–6], 11– [5]. Opioid-sparing multimodal analgesia protocols have been introduced to address these challenges, but their effectiveness in opioid tolerant patients remains inconsistent [8, 14–18]. Current dental guidelines do not specifically address postoperative pain management in opioid tolerant patients.

Patients receiving long-term opioid therapy represent a clinically recognisable group who may be at an increased risk of postoperative pain-related re-presentation following minor oral surgery. Whether this association reflects opioid tolerance itself or other patient and procedural factors remains uncertain. This study therefore sought to determine whether opioid exposure is associated with an increased risk of postoperative pain-related re-presentation requiring escalation of analgesia following minor oral surgery.

## Materials and methods

### Study design and setting

This study was conducted as a single-centre retrospective cohort study within the Oral Surgery Department of the Oral Health Service, Nepean Blue Mountains Local Health District (NBMLHD), New South Wales, Australia. The study evaluated postoperative pain outcomes following minor oral surgical procedures performed between May 2020 and May 2024.

Ethics approval was obtained from the NBMLHD Human Research Ethics Committee (Apollo Committee; Project Number: 2024APO_037). As this study involved retrospective analysis of de-identified clinical records, individual patient consent was not required.

### Indigenous status

Indigenous status was included as a routinely collected demographic variable in accordance with Australian public health reporting practices and was examined as a potential covariate rather than a primary exposure of interest.

### Data sources

Clinical data were retrieved from the electronic Oral Health Record system (Titanium) and the Cerner electronic Medical Record (eMR) PowerChart system. Data extraction was performed on NSW Health–approved secure systems, and all data were de-identified prior to analysis.

### Study population

#### Inclusion criteria

Records of patients who underwent minor oral surgical procedures were analysed. For the purpose of the study, simple and surgical dental extractions (removal of teeth requiring raising mucoperiosteal flap and removal of bone) were eligible for inclusion. Patients were classified as opioid tolerant if they met at least one of the following criteria at the time of surgery: prescribed opioid medications for longer than one month, receiving methadone maintenance therapy, self-reported current illicit opioid use and/or documented history of opioid use disorder. Despite differences in opioid exposure, these groups share opioid receptor adaptations that may reduce opioid responsiveness. Patients not meeting these criteria were classified as opioid-naïve and served as the control group.

#### Exclusion criteria

Patients were excluded if they met any of the following criteria: prescribed opioid medications for less than one month within the preceding six months, diagnosed with alveolar osteitis (dry socket) or secondary postoperative infection, incomplete clinical records preventing the assessment of postoperative analgesic protocol.

Postoperative pain management followed WHO guidelines [19] and current best protocols in Oral Surgery [1–3]. Patients were routinely advised to use Paracetamol 1 g and Ibuprofen 400 mg every 6 h postoperatively unless contraindicated. NSAIDs were avoided in patients with known contraindications, including hypersensitivity, asthma exacerbated by NSAIDs, renal impairment, or gastrointestinal pathology. Where patients were already receiving the maximum recommended daily dose of paracetamol or NSAIDs, additional dosing was not prescribed. Opioid medications were not routinely prescribed postoperatively and were reserved for management of uncontrolled postoperative pain when patients re-presented with inadequate pain control, as determined by the treating clinician. All simple and surgical extractions included for the study were performed under Local Anaesthetic (2% Lignocaine with 1:80000 Adrenaline). Due to limited number of patients undergoing procedures with long-acting agents in outpatient clinic setting and to maintain standardisation, no other local anaesthetic use was included in the study. All patients were provided with written post operative care instructions.

### Outcome measures

#### Primary outcome

The primary outcome that was measured was return presentation for uncontrolled post operative pain requiring escalation of analgesia following minor oral surgery. This outcome was selected to reflect clinically meaningful inadequate postoperative pain control in an outpatient oral surgery setting, where formal pain scoring is not routinely documented. A “return with pain” was defined as an unplanned postoperative presentation or documented clinical contact in which current postoperative analgesic pathways may not fully meet the needs of some opioid-exposed patients.

### Covariates and clinical variables

The following variables were extracted for analysis: age and gender, smoking and alcohol use (as documented in medical records), indigenous status (as self-reported in clinical records), type of extraction (simple vs. surgical) and current opioid medication and dose at the time of surgery. Opioid doses were converted to oral Morphine milligram Equivalent Daily Dose (oMEDD) using the Australian and New Zealand College of Anaesthetists (ANZCA) opioid dose equivalence Table [20]. Converting various opioid doses using oMEDD also allows for patients with different opioid exposures to be combined for statistical analysis. This study was conducted and reported in accordance with the Strengthening the Reporting of Observational Studies in Epidemiology (STROBE) guidelines for observational cohort studies.

### Statistical analysis

Descriptive statistics were used to summarise demographic and clinical characteristics. Categorical variables were expressed as frequencies and percentages, and continuous variables were expressed as means where appropriate. Comparisons between opioid tolerant and opioid naïve groups were performed using chi-square testing for categorical variables and Student’s t-tests for continuous variables.

Univariable and multivariable logistic regression analyses were performed to identify factors associated with postoperative re-presentation due to pain. The multivariable model included age, sex, Indigenous status, smoking/alcohol use, procedure type, opioid use for greater than one month, and recreational drug use/methadone exposure. Odds ratios (ORs) and adjusted odds ratios (aORs) with 95% confidence intervals (CIs) were reported. Statistical significance was defined as *p* < 0.05. All analyses were conducted using JASP and GraphPad Prism version 10.6.1. A post hoc power analysis was performed using G*Power version 3.1.9.7 to assess the power of the study to detect the observed difference between the groups.

## Results

### Study population

A total of 1263 patient records met the inclusion criteria and were included in the analysis. Of these, 184 patients (14.6%) were classified as opioid tolerant and 1079 patients (85.4%) were opioid naïve. The demographic characteristics of the study population are summarised (Table 1). The proportion of males was similar between opioid tolerant (61.95%) and opioid naïve (63.11%) groups. The mean age was comparable between groups (opioid tolerant: 51.2 years; opioid naïve: 51.16 years). Opioid tolerant patients demonstrated higher rates of smoking and alcohol use compared with opioid naïve patients (28.8% vs. 6.95%), *p* < 0.001. The proportion of Indigenous patients observed in the opioid tolerant group (23.36%) was comparable with the opioid naïve group (27.61%). Interestingly, simple extraction was significantly higher in the opioid tolerant (11.4% vs. 6.5%), *p* < 0.05. Surgical extractions accounted for the majority of procedures in both groups but were significantly higher in the opioid naïve group, *p* < 0.05.

**Table 1:** Demographic and clinical characteristics of the study population

	Gender (%)		Age (Mean) Years		Return with pain (%)		Smoking/Alcohol (%)		Identifying as Indigenous (%)	Type of extraction (%)			
	Males	Females	Males	Females						Simple		Surgical	
Opioid Tolerant *n* = 184	114 (61.95)	70 (38.04)	51.2	51.11	53 (28.80)	*P* < 0.001	53 (28.8)	*P* < 0.001	43 (23.36)	21 (11.41)	*P = 0.017*	163 (88.58)	*P = 0.017*
Opioid Naïve *n* = 1079	681 (63.11)	398 (36.88)	51.16	50.08	24 (2.22)		75 (6.95)		298 (27.61)	70 (6.48)		1009 (93.51)	

### Primary outcome

#### Opioid tolerance and recreational drug use

Return with postoperative pain requiring additional opioid analgesia occurred in 53 of 184 opioid tolerant patients (28.80%), compared with 24 of 1079 opioid naïve patients (2.22%) (Table 1). A post hoc power analysis demonstrated 100% power to detect the observed difference between groups at a two-sided α level of 0.05.

In univariable logistic regression analysis, age, sex, Indigenous status, and procedure type were not significantly associated with postoperative opioid-related re-presentation. In contrast, smoking/alcohol use (OR 3.24, 95% CI: 1.86–5.63; *p* < 0.001), opioid tolerance (OR 17.78, 95% CI: 10.62–29.77; *p* < 0.001), and recreational drug use or methadone exposure (OR 30.27, 95% CI: 13.58–67.46; *p* < 0.001) were significantly associated with an increased likelihood of re-presentation requiring additional opioid analgesia.

After multivariable adjustment, smoking/alcohol use was no longer associated with postoperative opioid-related re-presentation (aOR 0.83, 95% CI: 0.42–1.67; *p* = 0.604). Age, sex, Indigenous status, and procedure type also remained non-significant. In contrast, opioid tolerance (aOR 24.59, 95% CI: 13.26–45.62; *p* < 0.001) and recreational drug use/methadone exposure (aOR 59.28, 95% CI: 21.81-161.14; *p* < 0.001) remained strong independent predictors of postoperative opioid-related re-presentation. Notably, the magnitude of these associations increased after adjustment, suggesting that the observed effects were not explained by demographic characteristics, smoking/alcohol use, or procedure type. The subgroup analysis of postoperative pain outcome is reported in Table 2.

**Table 2:** Univariable and multivariable logistic regression analysis of factors associated with postoperative opioid-related re-presentation

Variable	Unadjusted OR (95% CI)	*p*-value	Adjusted OR (95% CI)	*p*-value
Age (per year increase)	1.007 (0.995–1.018)	0.246	1.011 (0.998–1.024)	0.111
Male sex	0.92 (0.57–1.47)	0.721	1.06 (0.61–1.86)	0.837
Indigenous status	0.64 (0.36–1.14)	0.128	0.52 (0.26–1.05)	0.069
Smoking/alcohol use	3.24 (1.86–5.63)	< 0.001	0.83 (0.42–1.67)	0.604
Simple vs. surgical extraction	1.10 (0.46–2.59)	0.837	0.71 (0.26–1.98)	0.518
Opioid tolerance	17.78 (10.62–29.77)	< 0.001	24.59 (13.26–45.62)	< 0.001
Recreational drug use/methadone	30.27 (13.58–67.46)	< 0.001	59.28 (21.81-161.14)	< 0.001

### Gender

Within the opioid tolerant cohort, postoperative re-presentation requiring additional opioid analgesia occurred in 30.70% of males and 27.14% of females. The proportion of males and females was similar between the opioid-tolerant and opioid-naïve groups (61.9% vs. 63.1% and 38.0% vs. 36.9%, respectively; *p* > 0.05). Sex was not significantly associated with postoperative opioid-related re-presentation requiring additional opioid analgesia in multivariable analysis (Table 2).

### Type of extraction

Among the 53 opioid tolerant patients who returned with postoperative pain requiring additional opioid analgesics, simple extractions accounted for a return rate of 23.80% (*n* = 5), while surgical extractions accounted for 29.99% (*n* = 48). In contrast, opioid‑naïve patients demonstrated no postoperative pain returns following simple extraction (*n* = 0), whereas all postoperative pain presentations occurred following surgical extractions (2.38%, *n* = 24). No correlation between complexity of extraction and return with post operative pain requiring additional opioid analgesics were observed (Table 2).

### Indigenous status

Among opioid-tolerant Indigenous patients, postoperative re-presentation requiring additional opioid analgesia occurred in 25.9% (*n* = 7) of males and 6.3% (*n* = 1) of females. In the opioid-naïve cohort, only one Indigenous male patient re-presented with postoperative pain, with no cases among Indigenous females. Indigenous status was not significantly associated with postoperative re-presentation requiring additional opioid analgesia in multivariable analysis (aOR 0.52, 95% CI: 0.26–1.05; *p* = 0.069).

### Oral morphine milligram equivalent dose

**Fig. 1:**
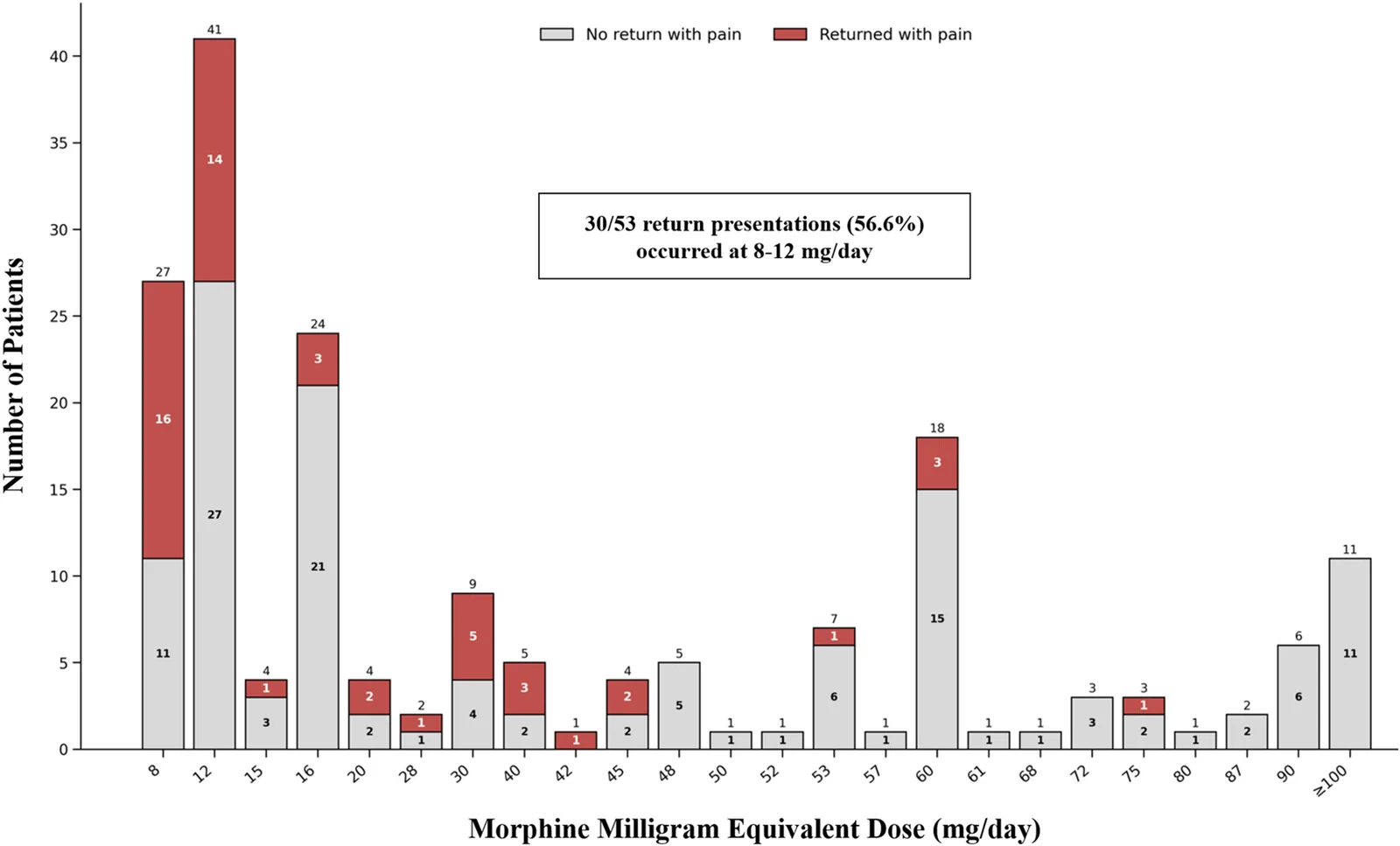
Distribution of Oral Morphine Equivalent Daily Dose (oMEDD) among opioid-tolerant patients with and without postoperative pain-related re-presentation

**Fig. 2:**
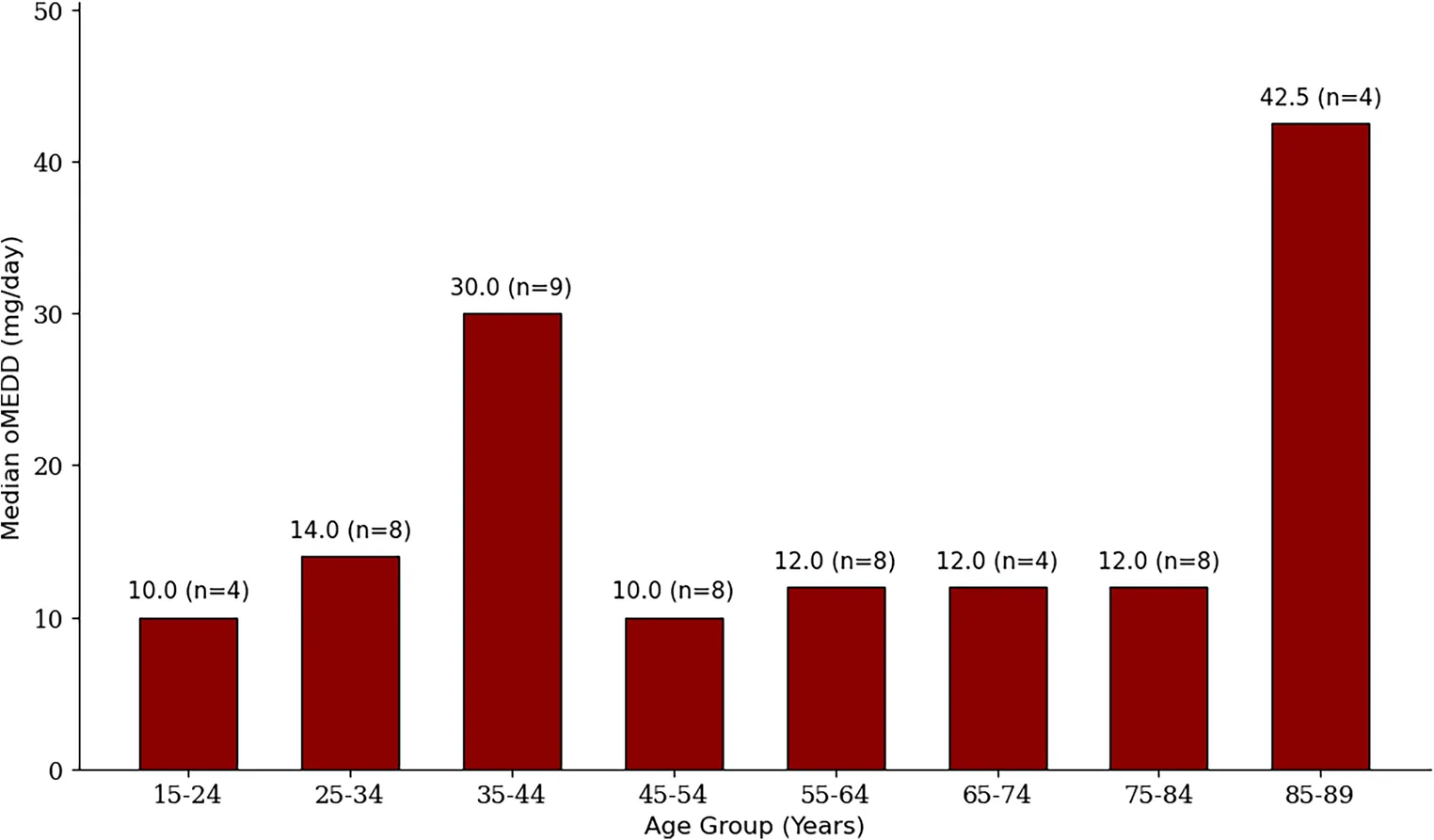
Median Oral Morphine Equivalent Daily Dose (oMEDD) by age group among opioid-tolerant patients requiring additional opioid analgesia

Opioid medications prescribed at the time of surgery were converted to oral morphine milligram equivalent daily dose (oMEDD). Among opioid tolerant patients who returned with postoperative pain requiring additional opioid analgesics (*n* = 53), the highest frequency of return (30 patients) was observed in patients receiving lower oMEDD values (less than or equal to 12 mg/day), corresponding to weak opioid regimens such as paracetamol–codeine combinations (Fig. 1). Median oMEDD in this group was significantly lower than in opioid-tolerant patients who did not return with pain (*n* = 131) (≤ 12 mg/day vs. 30 mg/day, *p* < 0.05). Age-stratified analysis of patients requiring additional opioid analgesia demonstrated variation in median oMEDD across age groups (Fig. 2). The largest number of patients were aged 35–44 years (*n* = 9), while the highest median oMEDD was observed among patients aged 85–89 years (42.5 mg/day). These findings are descriptive and should be interpreted cautiously given the small number of patients in several age groups.

## Discussion

This retrospective cohort study demonstrates that opioid tolerant patients undergoing minor oral surgery have a significantly higher rate of unplanned returns to the clinic indicating they are at a substantially higher risk of inadequate postoperative pain control when managed using standard analgesic protocols developed for opioid naïve patients. Notably, adjustment strengthened the observed associations, with the odds ratio for opioid tolerant increasing from 17.78 to 24.59 and the odds ratio for recreational drug use/methadone increasing from 30.27 to 59.28, suggesting that these factors were independently associated with postoperative opioid-related re-presentation and were not explained by demographic or procedural characteristics. Nearly one-third of opioid tolerant patients returned with postoperative pain requiring additional opioid analgesia, compared with just over 2% of opioid naïve patients (Table 2). This marked disparity persisted across demographic subgroups and procedural complexity, highlighting opioid tolerance as a dominant risk factor for poor postoperative pain outcomes in the outpatient oral surgery setting.

The findings of this study align with existing evidence from non-dental surgical disciplines demonstrating that opioid tolerance is associated with increased re-presentation requiring additional opioid analgesia [4–6, 11, 12]. The magnitude of the difference observed in this study is notable and suggests that analgesic strategies effective in opioid naïve dental patients may be insufficient in opioid tolerant individuals.

Opioid tolerance alters nociceptive processing through multiple mechanisms, including receptor downregulation and opioid-induced hyperalgesia [8, 9]. These changes may reduce responsiveness not only to opioids but also to non-opioid analgesics, limiting the effectiveness of standard postoperative regimens relying primarily on paracetamol and NSAIDs. The present findings suggest that reliance on these regimens alone may fail to address the altered pain physiology of opioid tolerant patients following oral surgery.

### Implications for opioid-sparing multimodal analgesia

Opioid-sparing multimodal analgesia protocols have been widely promoted to reduce opioid exposure while maintaining adequate pain control. In dentistry, this approach has been particularly successful in opioid naïve populations [2, 3]. However, the results of this study suggest that such protocols, when applied without modification, may be insufficient for opioid tolerant patients.

While multimodal analgesia remains an appropriate foundation for postoperative pain management, opioid tolerance may necessitate additional strategies. These may include preoperative identification of opioid tolerant patients, individualised analgesic planning, and consideration of adjunctive agents such as gabapentinoids or other non-opioid modalities supported by evidence in other surgical contexts [14–16]. Importantly, these findings do not support routine escalation of opioid prescribing but rather underscore the need for tailored approaches that balance effective analgesia with opioid stewardship.

### Weak opioids and uncontrolled postoperative pain

The data in this study demonstrates the higher frequency of postoperative return among patients receiving lower oral morphine milligram equivalent doses, particularly those prescribed weak opioid combinations such as paracetamol–codeine. This observation is consistent with evidence questioning the analgesic efficacy of weak opioids in acute postoperative pain and supports the view that such agents may offer limited benefit in opioid tolerant patients [3, 21]. While this finding should be interpreted cautiously, it reinforces the need to critically evaluate the role of weak opioids in postoperative pain management for this population.

### Demographic and clinical factors

Smoking and alcohol use were more prevalent among opioid tolerant patients, consistent with previous studies linking substance use to increased postoperative pain and analgesic requirements [22, 23]. These factors may act as confounders and contribute to heightened pain experiences independent of opioid tolerance. However, the persistence of increased postoperative pain across subgroups suggests that opioid tolerance itself plays a central role.

The low proportion of Indigenous female patients within both the opioid tolerant and opioid naïve cohorts in this study warrants careful consideration. While the study was not designed to investigate causal pathways underlying this observation, it highlights the importance of culturally sensitive and equitable approaches to pain management. Interpretation of subgroup analyses involving Indigenous patients is limited by small sample sizes, and these findings should not be overgeneralised. Nonetheless, they underscore the need for further research addressing disparities in pain management outcomes.

The findings of this study have important implications for oral surgical practice. Routine postoperative analgesic protocols designed for opioid naïve patients may not adequately address the needs of opioid tolerant individuals. Preoperative identification of opioid tolerance should prompt consideration of individualised pain management strategies and closer postoperative follow-up.

Future research should focus on prospective studies evaluating tailored multimodal analgesia protocols in opioid tolerant dental patients, including the role of adjunctive agents and non-pharmacological strategies. Randomised controlled trials are needed to establish evidence-based guidelines specific to this population and to determine optimal approaches that balance effective pain control with responsible opioid prescribing.

### Strengths and limitations

A key strength of this study is the large sample size relative to existing literature in this field, particularly within oral surgery. The use of real-world clinical outcomes provides pragmatic insight into postoperative pain management in routine practice. Several limitations must be acknowledged. The retrospective design precludes direct measurement of pain severity using validated pain scales and relies instead on return with pain requiring additional opioid analgesia as a surrogate outcome. While this outcome reflects clinically meaningful inadequate pain control in outpatient practice, it may underestimate the true prevalence of postoperative pain.

Although the study identified patients who re-presented with postoperative pain through Emergency Department electronic medical records and self-reported General Practitioner visits, the actual number of affected patients may be higher. Patients who did not disclose additional opioid use, sought care elsewhere, or simply endured pain without seeking medical attention would not have been captured by the study.

An additional limitation relates to the heterogeneous nature of opioid exposure within the opioid-tolerant patients; however, the consistency of findings across demographic and procedural subgroups suggests that this is unlikely to fully explain the observed differences.

Although oral morphine equivalent daily dose (oMEDD) calculations were used to standardize opioid exposure, variations in opioid type, indication, duration of use, and prescribing patterns may have introduced residual confounding that could not be fully accounted for in the analysis.

The absence of long-term follow-up limits assessment of sustained opioid use or longer-term pain outcomes. Furthermore, while exploratory analyses were performed, the study was not powered to detect differences within smaller subgroups.

## Conclusion

This study demonstrates that opioid tolerant patients undergoing minor oral surgery are at increased risk of inadequate postoperative pain control when managed with standard protocols for opioid naïve patients. Nearly one-third required additional opioid analgesia compared with a small proportion of opioid naïve patients. Opioid exposure emerged as a clinically relevant marker associated with higher re-presentation rates. While opioid-sparing multimodal analgesia remains important, it may be insufficient without modification in this group. Recognition of opioid tolerance during preoperative assessment and tailored postoperative pain management are essential to improve outcomes while maintaining responsible opioid stewardship. Further prospective studies are needed to establish evidence-based guidelines.

## Data Availability

The data that support the findings of this study are available from Nepean Blue Mountain Local Health District, but restrictions apply to the availability of these data, which were used under license for the current study, and so are not publicly available. Data are, however, available from the authors upon reasonable request and with permission of Nepean Blue Mountain Local Health District.
